# Supplementary material for: How internet use has transformed educational expectations in Chinese families
Source: PLoS One. 2025 Apr 29;20(4):e0321185. doi: 10.1371/journal.pone.0321185 (PMC12040204; doi:10.1371/journal.pone.0321185)
Supplement: Attachment — Submitted filename: Response.docx [file pone.0321185.s003.docx]

**Response to Reviewers' Comments**

Manuscript ID: PONE-D-24-23805

**How Internet Use Has Transformed Educational Expectations in Chinese Families**

Thank you for the opportunity to revise and resubmit the manuscript (PONE-D-24-23805 ) for Plos One. We sincerely appreciate the reviewers' comments. We consider all the comments an opportunity to improve the manuscript. We have now addressed all the suggested comments. The detailed response is tabulated in the following section. We have marked all the revised parts in red for your convenience.

**Reviewer 1**

This paper addressed an interesting question: why parents hold high education expectations in China (even for those from low SES backgrounds)? As we know, low SES parents in other countries like U.S. usually maintain low education expectations instead. The authors argued that internet use might be a reason. I have several comments on this paper.

***Response:***

***We would like to thank you for your very positive feedback and are grateful to you for your constructive and insightful comments. We have addressed all your comments and now outline our point-by-point responses below.***

**Reviewer 1 - Comment 1**

The authors should elaborate more on how and why internet use affect education expectations, particularly in Literature Review and Heterogeneity analysis. Also, the measurement is "the spare time for internet", but I think it is necessary to differentiate types of usage, like whether they use internet for Tik-tok, self-learning or business. The second-level and third-level of digital divide has argued the importance of usage and gain via internet. (The authors may also add some literature on digital divide.) If CFPS lacks such measurement, the authors should explain it and admit such limitation.

***Response 1:***

***Thank you for this specific comment. We have made three modifications: First, we further analyzed the impact of different Internet use purposes (Study, Work, Business, Social intercourse, Entertainment) on educational expectations in the empirical study; Secondly, we have added literature and theories on the digital divide and educational expectations in the literature review and theoretical hypothesis sections; Thirdly, we discussed the limitations of the research based on the three levels of digital divide.Please see below for what we have revised:***

***In sec*tion Abstract (on page: 1):**

… ' Besides, only using the Internet for non-entertainment purposes can promote parents' educational expectations of their children. '…

***In sec*tion 2.1 The digital divide and educational expectations (on pages: 4-6):**

2.1 The digital divide and educational expectations

Recent advancements in digital technologies—centered around mobile Internet, cloud computing, and big data—have profoundly transformed economic and social development, as well as individual production and daily life. However, in the process of popularizing digital technology, significant disparities have emerged among social groups regarding access to information, the ability to acquire it, and the benefits derived from it. These differences have evolved into what is known as the "digital divide" (17).

The digital divide has a dual impact on the educational expectations of both parents and children. On one hand, unequal access to digital technology directly affects the distribution of educational resources. Viaene and Zilcha (18) argue that this inequality influences students' ability to engage with online educational resources. As digitalization progresses, an increasing number of high-quality educational resources—such as online courses, educational software, and e-books—are disseminated via the Internet. However, the digital divide prevents these resources from being equally available to all students. Urban students from affluent families are more likely to access and leverage these resources, while students from rural or economically disadvantaged backgrounds often lack the necessary technological support. This disparity in access to digital resources contributes to an educational disadvantage, which ultimately lowers the educational expectations of disadvantaged student groups.

On the other hand, the digital divide contributes to a broader 'family education divide' (19). Families with higher economic capital can capitalize on their digital advantages to quickly acquire the latest and high-quality educational information, allowing them to set ambitious educational expectations for their children and support these expectations with relevant information and modern educational approaches. Conversely, families disadvantaged by the digital divide struggle to access this information and must rely on outdated or limited resources and traditional educational concepts. The resulting information asymmetry exacerbates inequalities, influencing the level and structure of these families' educational expectations for their children (20).

While the existing literature provides indirect support for the notion that the Internet impacts educational expectations through the digital divide, it lacks direct evidence with broad, policy-relevant implications.

***In sec*tion 4.3 Educational expectations under different Internet use purposes (on pages: 17-19):**

**4.3 Educational expectations based on different Internet use purposes**

We further examine how different online activities influence educational expectations. The CFPS database includes a survey that asks individuals about the frequency of their Internet use for various purposes, including learning, work, business, socializing, and entertainment. Respondents' frequency of use is rated on a scale: almost every day=7, 3-4 times a week=6, 1-2 times a week=5, 2-3 times a month=4, once a month=3, once every few months=2, and never=1. The results are presented in Table 5.

Our findings reveal that when parents use the Internet for learning and work, their educational expectations for their children are the highest, followed by business activities and social networking. As expected, frequent online entertainment by parents does not significantly enhance their expectations for their children's education. Compared to parents who primarily engage in recreational activities online, those who use the Internet for studying, working, and conducting business are more likely to gain knowledge and self-improvement, which reinforces their focus on their children's education. These results indicate that the purpose of Internet use can significantly impact parents' educational expectations.

**Table 5** The Purpose of Internet Use and Educational Expectations

| VARIABLES | (1) | (2) | (3) | (4) | (5) |
| --- | --- | --- | --- | --- | --- |
|  | Logit | Logit | Logit | Logit | Logit |
| ***Study*** | 0.1211^***^ |  |  |  |  |
|  | (0.0197) |  |  |  |  |
| ***Work*** |  | 0.1170^***^ |  |  |  |
|  |  | (0.0208) |  |  |  |
| ***Business*** |  |  | 0.1015^***^ |  |  |
|  |  |  | (0.0226) |  |  |
| ***Social intercourse*** |  |  |  | 0.0825^***^ |  |
|  |  |  |  | (0.0237) |  |
| ***Entertainment*** |  |  |  |  | -0.0002 |
|  |  |  |  |  | (0.0219) |
| *Child’s gender* | -0.0521 | -0.1852^*^ | -0.0523 | -0.0522 | -0.0554 |
|  | (0.0831) | (0.0968) | (0.0830) | (0.0830) | (0.0828) |
| *Child’s age* | -0.0562^***^ | -0.0637^***^ | -0.0569^***^ | -0.0578^***^ | -0.0582^***^ |
|  | (0.0103) | (0.0118) | (0.0104) | (0.0104) | (0.0103) |
| *Parental gender* | -0.3795^***^ | -0.3919^***^ | -0.3288^***^ | -0.3211^***^ | -0.3222^***^ |
|  | (0.0896) | (0.0992) | (0.0887) | (0.0892) | (0.0887) |
| *Parental age* | 0.0195^***^ | 0.0167^**^ | 0.0207^***^ | 0.0180^***^ | 0.0141^**^ |
|  | (0.0060) | (0.0070) | (0.0060) | (0.0061) | (0.0060) |
| *Household register* | -0.8235^***^ | -0.8496^***^ | -0.8460^***^ | -0.9107^***^ | -0.9389^***^ |
|  | (0.1240) | (0.1534) | (0.1242) | (0.1216) | (0.1216) |
| *Income* | 0.0268 | 0.0716 | 0.0359 | 0.0439 | 0.0468 |
|  | (0.0377) | (0.0499) | (0.0374) | (0.0377) | (0.0377) |
|  | 0.1728^**^ | 0.2034^**^ | 0.1610^*^ | 0.1534^*^ | 0.1563^*^ |
|  | (0.0847) | (0.0982) | (0.0846) | (0.0845) | (0.0844) |
| *Family size* | -0.0047 | 0.0031 | -0.0050 | -0.0070 | -0.0046 |
|  | (0.0192) | (0.0226) | (0.0190) | (0.0191) | (0.0191) |
| Area Control | $\surd$ | $\surd$ | $\surd$ | $\surd$ | $\surd$ |
| Constant | 1.7514^***^ | 1.8795^***^ | 1.6816^***^ | 1.7130^***^ | 2.3507^***^ |
|  | (0.3032) | (0.3706) | (0.3265) | (0.3433) | (0.3288) |
| Observations | 4,587 | 3,447 | 4,588 | 4,585 | 4,586 |

*Note*: Robust standard errors in parentheses; ^***^ *p*<0.01, ^**^ *p*<0.05, ^*^ *p*<0.1； The deletion of samples that answered 'I don't know' or were missing resulted in the missing sample size in Table 5.

***In sec*tion 5.1 Summary (on pages: 22-25):**

… ' Our research results emphasize that accessing the Internet via mobile phones or computers can affect parents' expectations of children's education. Nevertheless, it must be for non-entertainment purposes. '…

… ' That said, this study has several limitations. First, the digital divide encompasses three levels: "access gap," "usage gap," and "knowledge gap." However, due to data constraints, this study primarily focuses on the impact of "access channels" on educational expectations, leaving the other dimensions unexplored.'…

**Reviewer 1 - Comment 2**

Hypothesis 2, the social interaction mechanism, needs revision. If the authors wanted to test the mechanism, they'd better use mediation analysis. Currently, we only see the correlation between internet use and social interaction in Table 6, yet how social interaction, particularly the measurements in this paper, affect education expectations is not discussed.

***Response 2:***

***Thank you very much for this comment.*** ***We further strengthened the measurement of social interaction and reexamined the mechanism. Please see below for what we have revised:***

***In sec*tion 3.2.3 Mediator Variable (on page: 10):**

***Social interaction:*** There is a strong positive correlation between social gift expenditures and the level of social interaction. In traditional Chinese society, the practice of "reciprocal gift-giving" underscores the importance of social relationships. By exchanging gifts, individuals express appreciation, gratitude, and care, thereby maintaining and strengthening social bonds. As social interaction increases, individuals may incur higher expenses on gifts and favors to sustain these relationships. Since family values primarily influence offline social interactions, we measure the level of social interaction by the amount spent on social gifts, as reported in the questionnaire.

***In sec*tion 4.5 Mechanism testing (on pages: 21-22):**

Given that the Logit regression model is a nonlinear probability model, this study utilizes the Kaplan Meier with Breslow (KHB) method for tiered data to test the mediating effects within such models. The KHB method decomposes the results into three components: the estimation results of the simplified (Reduced) model, the full (Full) model, and the difference (Diff) between the two. Moreover, the simplified model represents the total effect, while the full model represents the direct effect, and the difference between the two reflects the mediating effect.

Table 7 presents the decomposition results of the mediating effect of social interaction on the relationship between Internet use and educational expectations. The findings indicate that the estimated coefficient of the difference model is 0.0005, significant at the 1% level, suggesting that social interaction mediates the positive impact of Internet use on educational expectations. This result implies that Internet use enhances parents' social interactions, and through these interactions, parents acquire more information (shaping their cognition) or raise their educational expectations for their children, driven by comparative psychology.

**Table 7** Internet Use for Educational Expectations Test - Social Interaction Effects

| Model | Coefficient | Standard error | Z value | P value |
| --- | --- | --- | --- | --- |
| Reduced Model | 0.0103 | 0.0035 | 2.96 | 0.003 |
| Full Model | 0.0097 | 0.0035 | 2.81 | 0.005 |
| Diff Model | 0.0005 | 0.0002 | 2.52 | 0.012 |

**Reviewer 1 - Comment 3**

I would like to propose another comment on the theoretical framework. We usually use Confucian culture to explain the high education expectations of the Chinese. Yet if the high education expectations are driven by internet use, how can we explain the high education expectations before the wide access to internet since 1990s? I think the authors should reconsider this question and revise the Introduction accordingly.

***Response 3:***

***Thank you very much for this comment. We have revised the introduction, especially emphasizing the reasons for high education expectations before the advent of the Internet. Please see below for what we have revised:***

***In sec*tion Introduction (on pages: 1-2):**

… ' In the 1980s, Bourdieu (4) introduced the theory of "cultural reproduction" to explain the disparities in educational expectations among families. Based on this theory, parents with high cultural capital use cultural reproduction as a means of achieving social reproduction, thereby preserving their elite status and identity as cultural symbols.

Furthermore, Bourdieu's theory laid the foundation for subsequent research (5-8) that analyzed the varying expectations parents have for their children's education based on innate factors such as education level, occupation, and income. These studies explore the reasons why family background influences educational expectations, particularly through the provision of knowledge and material resources for further education. However, they fall short in explaining why some families with lower socio-economic status still maintain high educational aspirations for their children.

In the context of Asia, particularly China, Confucianism offers a partial explanation. In Confucian culture, education is closely linked to social status, and the ancient imperial examination system, which served as the primary means of selecting officials, reinforced the notion that education could transform family and social status. Although the imperial examination system no longer exists, the belief that disadvantaged families can achieve social mobility through education persists.

This analysis suggests that parental cognition may play a more critical role than socio-economic background in shaping educational expectations. '…

**Reviewer 2**

The paper provides a comprehensive overview of the research area and is well-structured. This is an interesting study and the authors have used a unique dataset and cuttingedge methodology to demonstrate it. However, the paper can be enhanced by addressing the following aspects

***Response:***

***We would like to thank you for your very positive feedback and are grateful to you for your constructive and insightful comments. We have addressed all your comments and outlined our point-by-point responses below.***

**Reviewer 2 - Comment 1**

Longitudinal Data and Cross-Sectional Analysis: The China Family Panel Studies (CFPS) data used in this article is longitudinal, meaning it follows the same individuals over time. However, in your study, the authors pooled data from different waves (2016, 2018, and 2020) and treated it as cross-sectional data. This approach has limitations because the measuremts are not independent of each other in different waves.

To improve your analysis, consider either:Using a specific round of data (e.g., 2016) for cross-sectional analysis.Implementing panel data analysis to account for the three waves of data and individual-level changes.

***Response 1:***

***Thank you for this specific comment. We used data from 2018 for cross-sectional analysis and made changes to the research results of the entire article, while the research conclusions remained largely unchanged. See below for what we have added:***

***In section 3.1* Data sources *(on page: 9)*:**

… ' We selected 2018 survey data for our analysis from the available options. Compared to 2016, Internet development in 2018 was more advanced, with greater integration into people's daily lives. Additionally, to avoid the potential influence of the COVID-19 pandemic, we excluded data from 2020.

***In section 3.2.3 Control variable* *(on page: 11)*:**

**Table 1** Descriptive statistical analysis of variables

| **Categorization** | **Variable name** | **Variable Definition** | **N** | **Mean** | **Std. Dev.** |
| --- | --- | --- | --- | --- | --- |
| Dependent variable | *Edu expectation* | Expected years of education | 7,110 | 0.8280 | 0.3770 |
| Independent variable | *Internet use* | Internet usage time | 7,110 | 8.5850 | 11.5470 |
| Personal characteristics | *Child’s gender* | Male=1, Female=0 | 7,110 | 0.5290 | 0.4990 |
|  | *Child’s age* | Age value | 7,110 | 7.3620 | 4.3780 |
|  | *Parental gender* | Male=1, Female=0 | 7,110 | 0.3370 | 0.4730 |
|  | *Parental age* | Age value | 7,110 | 41.1370 | 12.5920 |
| Family characteristics | *Household register* | Agricultural household = 1, non-agricultural household = 0 | 7,110 | 0.8080 | 0.3940 |
|  | *Income* | Where does your personal income belong in your local area? 1~5 rising | 7,110 | 2.7860 | 1.1310 |
|  | *Residence* | Rural = 1, Urban = 0 | 7,110 | 0.5580 | 0.4970 |
|  | *Family size* | Number of family members | 7,110 | 5.4570 | 2.1630 |
| Mediator Variable | *Social interaction* | Log (Expenditures on gifts of favors +1) | 7,005 | 7.3127 | 2.3182 |

*Note*: Robust standard errors in parentheses; ^***^ *p*<0.01, ^**^ *p*<0.05, ^*^ *p*<0.1.

**Reviewer 2 - Comment 2**

Model Consistency and Terminology:In Section 3.3, the authors describe the benchmark model as a logit model (p17), but the header in Table 2 (P19) refers to it as probit. Consistency is crucial.Additionally, the header in Column (4) of Table 2 should be “odds ratio,” not “rate.” (P19) It should be noticed that ratios compare homogeneous indicators, while rates compare heterogeneous indicators.

***Response2:***

***Thank you for this specific comment. We have corrected the wording errors by replacing probit with logit and changing "odd rate" to "odd ratio". See below for what we have added:***

***In section 4.1 Baseline results* *(on page: 13)*:**

**Table 2** Benchmark regression results

| VARIABLES | (1) | (2) | (3) | (4) |
| --- | --- | --- | --- | --- |
|  | Logit | Logit | Logit | Odd Ratio |
| ***Internet use*** | **0.0141***** | **0.0143***** | **0.0111***** | **1.0111^***^** |
|  | **(0.0032)** | **(0.0037)** | **(0.0036)** | **(0.0036)** |
| *Child’s gender* |  | 0.0006 | 0.0030 | 1.0030 |
|  |  | (0.0634) | (0.0638) | (0.0640) |
| *Child’s age* |  | -0.0562*** | -0.0556*** | 0.9459^***^ |
|  |  | (0.0075) | (0.0076) | (0.0071) |
| *Parental gender* |  | -0.2423*** | -0.2659*** | 0.7665^***^ |
|  |  | (0.0673) | (0.0678) | (0.0519) |
| *Parental age* |  | 0.0083*** | 0.0078** | 1.0078^**^ |
|  |  | (0.0031) | (0.0031) | (0.0031) |
| *Household register* |  |  | -0.8159*** | 0.4422^***^ |
|  |  |  | (0.1012) | (0.0448) |
| *Income* |  |  | -0.0204 | 0.9798 |
|  |  |  | (0.0288) | (0.0282) |
| *Residence* |  |  | 0.0738 | 1.0766 |
|  |  |  | (0.0654) | (0.0704) |
| *Family size* |  |  | -0.0048 | 0.9952 |
|  |  |  | (0.0146) | (0.0146) |
| Area Control | $\surd$ | $\surd$ | $\surd$ | $\surd$ |
| Constant | 1.5468^***^ | 1.7167*** | 1.4430^***^ | 11.7496^***^ |
|  | (0.0601) | (0.1522) | (0.1170) | (2.4625) |
| Observations | 7,110 | 7,110 | 7,110 | 7,110 |

*Note*: Robust standard errors in parentheses; ^***^ *p*<0.01, ^**^ *p*<0.05, ^*^ *p*<0.1.

**Reviewer 2 - Comment 3**

Robustness test between logit and probit:While logit and probit models are almost the same link functions, there is a very rough approximation between the coefficients of the probit and logit: multiply the probit coefficient by 1.8 to get logit coefficient, this can be traced back to the (pi/sqrt(3)) difference in the variances of the models. Therefore, the probit model in the first column of table 3 should not be used as the robustness test result of the logit model.(P21)

***Response3:***

***Thank you for this specific comment. We removed the probit model from the robustness test.***

**Reviewer 2 - Comment 4**

Heterogeneity Tests and Chow Test: When all heterogeneity test coefficients in Table 5 are significant (p25), it doesn’t necessarily mean one group’s coefficient is greater than another’s. To make such claims, consider using the Chow test. It assesses whether coefficients differ significantly between groups, providing more robust evidence of differential impacts.

***Response4:***

***Thank you for this specific comment. We used Seemingly Unrelated Regression (SUEST) to confirm that there were differences in coefficients between the groups. See below for what we have added:***

***In section 4.4 Heterogeneity analysis* *(on pages: 19-20)*:**

… ' To ensure robustness, we further tested for significant differences in coefficients between groups. The results of the Seemingly Unrelated Regression (SUEST) analysis revealed that the p-values for the differences in regression coefficients across groups were all less than 0.01, indicating significant variations in the regression coefficients between the groups.'…
