# Supplementary material for: How internet use has transformed educational expectations in Chinese families
Source: PLoS One. 2025 Apr 29;20(4):e0321185. doi: 10.1371/journal.pone.0321185 (PMC12040204; doi:10.1371/journal.pone.0321185)
Supplement: Attachment — Submitted filename: Review 10.docx [file pone.0321185.s005.docx]

Dear Editor,

I would like to express my gratitude for the opportunity to review the manuscript titled " How Internet Use Has Transformed Educational Expectations in Chinese Families." After a thorough evaluation, I have outlined several comments and suggestions below that aim to strengthen the overall quality and clarity of the manuscript:

1. While using social cognition theory is appropriate, the article should clarify how this theory specifically applies to the context of Chinese families and educational expectations.
2. The literature review is somewhat limited. It would benefit from a broader exploration of existing studies on the digital divide and educational expectations across different cultures, not just in China.
3. While the methodology is described, more detail is needed on how variables were operationalized, particularly "social interaction" and "Internet use intensity."
4. The authors should discuss potential biases in the CFPS data, such as response bias or sample representativeness, which could affect the findings.
5. The selection of control variables should be justified more thoroughly. Why were certain variables chosen, and how might omitted variables impact the results?
6. The statistical methods used (e.g., Logit model, IV-Probit) are appropriate, but the article should explain why these methods were chosen over others. Additionally, include a discussion on the assumptions underlying these models.
7. Some results, particularly in the regression tables, lack sufficient interpretation. The implications of significant coefficients should be discussed more explicitly in the text.
8. The discussion could be more critical. Address potential counterarguments or alternative explanations for the findings, especially regarding the influence of cultural factors on educational expectations.
9. While the recommendations are relevant, they could be more specific. For example, what concrete steps should policymakers take to improve Internet access and educational equity?
10. The limitations section mentions future research but could suggest specific areas for further investigation, such as longitudinal studies or comparative analyses with other countries.
11. The article would benefit from thorough proofreading to ensure consistency in formatting, especially in tables and figures. Clarity in presentation is crucial for reader comprehension.
12. Ensure that all tables and figures are referenced in the text and that their relevance is explained. Consider adding visual aids that summarize key findings for better engagement.
